# Post-developmental extracellular proteoglycan maintenance in attractin-deficient mice

**DOI:** 10.1186/s13104-020-05130-1

**Published:** 2020-06-24

**Authors:** Abdallah Azouz, Jonathan S. Duke-Cohan

**Affiliations:** 1 Department of Pathology, Regional One Health, University of Tennessee Health Science Center, Memphis, TN USA; 2 Department of Medical Oncology, Dana-Farber Cancer Institute, JF517, 450 Brookline Avenue, Boston, MA 02215 USA; 3 Department of Medicine, Harvard Medical School, Boston, MA USA

**Keywords:** Attractin, Extracellular matrix, Histology, Kidney, Liver, Proteoglycan

## Abstract

**Objective:**

Neurodegeneration and hair pigmentation alterations in mice occur consequent to aberrations at the *Atrn* locus coding for the transmembrane form of attractin. Earlier results pointed to a possible involvement in intracellular trafficking/export of secretory vesicles containing proteoglycan. Here we examined kidney and liver, both heavily dependent upon proteoglycan, of attractin-deficient mice to determine whether abnormalities were observed in these tissues.

**Results:**

Histological and histochemical analysis to detect glycosylated protein identified a severe loss in attractin-deficient mice of extracellular proteoglycan between kidney tubules in addition to a loss of glycosylated material within the intratubular brush border. In the liver, extracellular matrix material was significantly depleted between hepatocytes together with swollen sinuses and aberrations in the proteoglycan-dependent space of Disse. These results are consistent with a generalized defect in extracellular proteoglycan deposition in *Atrn*-mutant mice and support previous reports suggesting a role for attractin in the secretory vesicle pathway.

## Introduction

Attractin, initially discovered as a human secreted glycoprotein circulating at high concentrations in the periphery and enabling T cell-monocyte clustering [1], also exists as a transmembrane form produced by alternative splicing of the *ATRN* gene, while the mouse only produces the transmembrane form [2, 3]. On activated T cells, attractin moves in electron-dense proteoglycan-rich vesicles to the plasma membrane leading to transient extracellular expression [1, 4]. Mutations at the *Atrn* locus in the mouse result in the *mahogany* phenotype where, despite normal levels of the agouti protein that acts as an antagonist of α-melanocyte stimulating hormone (α-MSH), the agouti protein does not appear to be appropriately presented to the Melanocortin-1 receptor (Mc1R) and black/brown eumelanin synthesis persists rather than that of the lighter yellowish phaeomelanin [2]. Attractin’s role in agouti presentation remains to be fully elucidated. One possibility is that the membrane-anchored ectodomain may help present agouti protein by binding the positive N-terminal leaving the C-terminal free to interact with the Mc1R [5].

Attractin’s functional range has widened following reports that *mahogany* (*Atrn*^mg−3J/mg−3J^) mice present a juvenile-onset Central Nervous System (CNS)-confined neurodegeneration characterized by hypomyelination, axonal swelling, spongiform vacuolation and microtremors [5]. This neural phenotype is found not only in other mouse mutant *Atrn* alleles [6, 7] but also in the *zitter* and *myelin vacuolation* rats [8, 9], and the *black tremor* hamster [10], all now confirmed as *Atrn* mutants. The pigmentation phenotype and neuropathology are corrected in animals transgenic for membrane attractin [5, 8]. Embryonic development appears normal in *Atrn*-mutant mice; the neurodegeneration is manifest during juvenile maturation and may be related to a defect in maintaining the integrity of the plasma membrane with potentially severe consequences for oligodendrocyte-directed myelination [4, 11]. Attractin’s common biochemical role in immune cell interactions, regulation of pigmentation and neural pathology remains undefined. A function in vesicular transport of cargo, both proteoglycan and new lipid-raft rich membrane, to the plasma membrane is implicated.

## Main text

### Methods

#### Mice

Age-matched male C3HeB/FeJ mice and C3HeB/FeJ-*Atrn*^mg−3J/mg−3J^ homozygotes were obtained from the Jackson Laboratory (Bar Harbor, ME). Animals were housed maximum 3 to a cage according to institutional guidelines in an Association for Assessment and Accreditation of Laboratory Animal Care (AAALAC)-accredited facility at the Dana-Farber Cancer Institute. Since *Atrn* mutations are recessive, homozygous *Atrn*^mg−3J/mg−3J^ mice were mated with heterozygous *Atrn*^+/mg−3J^ mice resulting in litters where half the mice were wild-type phenotype and half the mice were recessive *Atrn* mutants. In any experiment, only control and mutant siblings from the same mating were compared (aged 3–3.5 months), and comparisons examined mice with no gender preference. Euthanasia was by CO_2_ inhalation followed by cervical dislocation with all procedures approved under Dana-Farber Cancer Institute Animal Care and Use Committee (ACUC) protocol 99-026.

#### Histology

Organs were excised and fixed in Bouin’s fluid, formalin, or methanol depending upon the subsequent staining protocol. Tissues were then embedded in paraffin and 4 μm sections were arranged on glass slides. Hematoxylin and eosin (H&E) staining followed standard histopathology procedures. For detection of glycoprotein, rehydrated sections were placed in periodic acid (0.5% in water) for 15 min, rinsed, placed in Schiff’s reagent (0.5% in water; Sigma, St Louis MO), developed in running water and counterstained with hematoxylin prior to mounting. Photomicroscopic images were digitally captured using the “Magnafire” system (Olympus, Melville NY).

#### Proteoglycan quantification

To quantitate relative proteoglycan staining using Periodic acid-Schiff reagent (PAS) staining in sections from control and attractin-deficient mice, histology images were imported as JPG files into ImageJ [12]. After setting the appropriate scale (in µm), the images were deconvoluted into red, blue and green (RGB) layers and converted into grey-scale images where the green layer gives the clearest distinction between PAS stain and background. Using the green layers, thresholds were set to exclude background signal, signal above threshold was identified and fractional representation of marked pixels in the image was recorded. For every paired analysis of tissues from control and attractin-deficient mice, identical image processing and set thresholds were used.

### Results

**Fig. 1:**
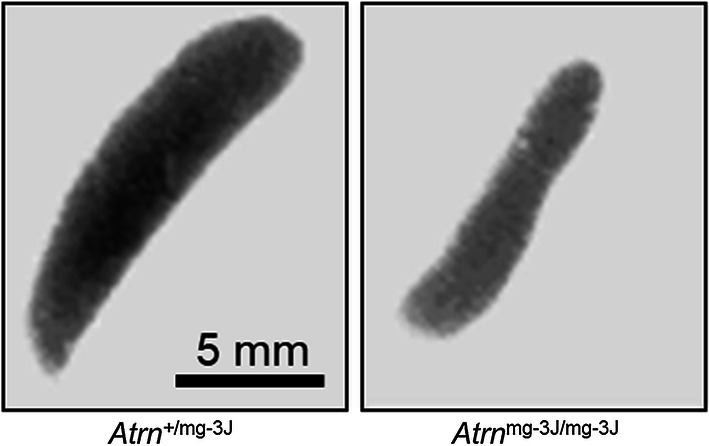
Spleens of older (~ 5 month) *Atrn*^mg−3J/mg−3J^ mice (right panel; × 3.5) are consistently half to two-thirds the size of age-matched controls (left panel; ×3.5), reflected also by mononuclear cell counts (wild-type: 6.17 × 10^7^ cells/spleen, *Atrn*-mutant: 3.9 × 10^7^ cells/spleen; pool of 3 for each genotype)

**Fig. 2:**
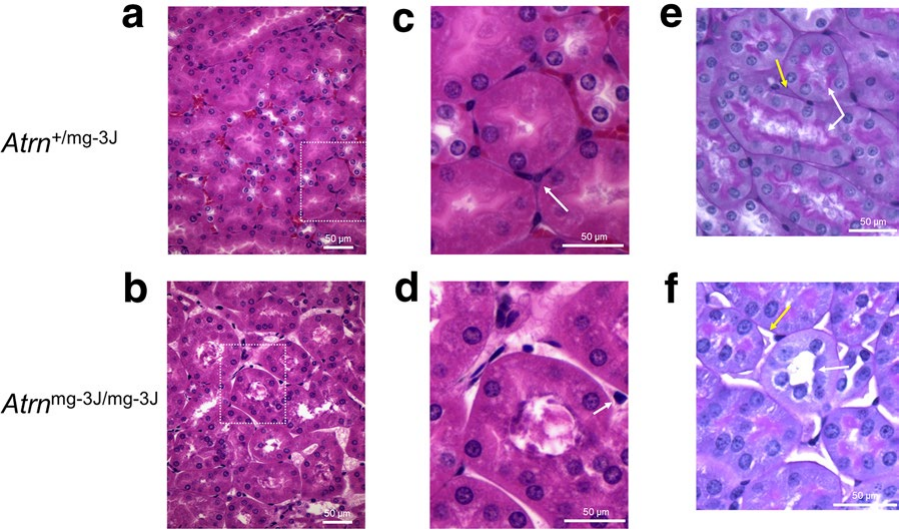
**a** Normal (*Atrn*^+/mg−3J^) kidney (H&E); **b***Atrn*^mg−3J/mg−3J^ kidney (H&E). Note in the normal condition the dense staining basement membrane surrounding each tubule which also forms a tight junction between the tubules. This staining is absent in the *Atrn*-mutant specimen. **c**, **d** Higher resolution images of images within insets (dotted lines) in **a**, **b**. Note that in both specimens there are interstitial cells between the tubules (arrows) but only in the normal section are these cells embedded in basement membrane. For all images, results are representative of more than 5 sections from 10 paired sibling comparisons. **e** Normal kidney; **f***Atrn*^mg−3J/mg−3J^ kidney (PAS stain). The intralumenal brush border of *Atrn*^mg−3J/mg−3J^ kidney tubules is depleted of extracellular matrix on comparison with control kidney (white arrows), and the intertubular space is similarly depleted (yellow arrows). For all images, results are representative of more than 5 sections from 10 paired sibling comparisons

**Fig. 3:**
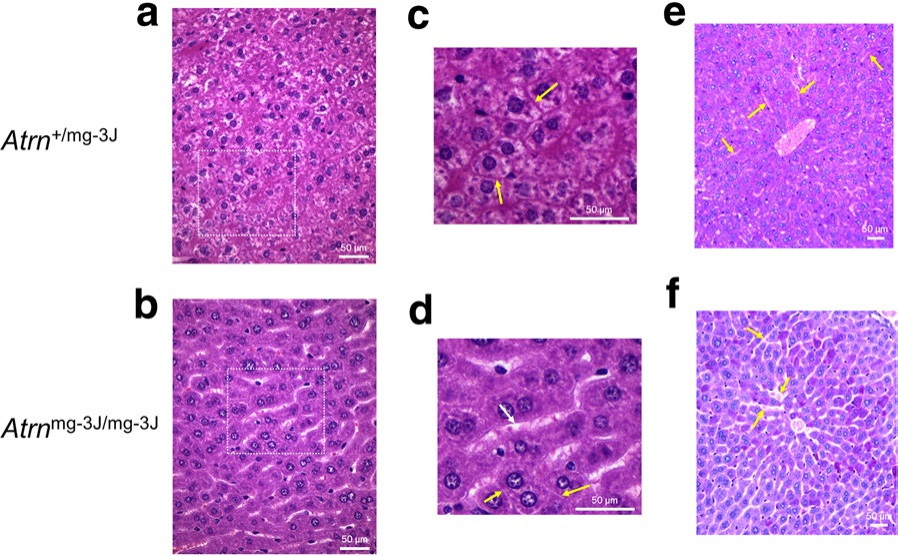
**a** Normal liver and **b***Atrn*^mg−3J/mg−3J^ liver (H&E stain). The sinusoids (white space between rows of hepatocytes) are readily apparent in the mutant mice. Glycogen does not stain with H&E leading to light areas within the control cells, an effect not seen in the mutant indicating depletion of glycogen. **c**, **d** Higher resolution images of images within insets (dotted lines) in **a**, **b**. White arrow identifies sinusoid, yellow arrows indicate intercellular space. **e** Normal liver; **f***Atrn*^mg−3J/mg−3J^ liver (PAS stain). Comparison of sinusoids (yellow arrows) clearly indicates presence of proteoglycan contributing to the perisinusoidal space of Disse in the control liver while such signal is absent in the attractin-deficient liver. Note also (at the yellow arrows) evidence for sinusoidal swelling in the *Atrn*^mg−3J/mg−3J^ liver accompanied by a more general lack of inter-hepatocyte ECM on comparison with control

In this report we demonstrate that attractin-deficient *mahogany* mice, despite apparently normal gross organ structure, have a severe juvenile-onset progressive loss of basement membrane within organs heavily dependent upon extracellular matrix (ECM) function. Our attention was drawn to overall organ structure by the consistent observation that the spleens of older *Atrn*^mg−3J/mg−3J^ mice (~ 5 months or more) are half to two-thirds the size of spleens from wild-type or heterozygous littermates (Fig. 1). Since the neuropathology in *Atrn*-null mice is moderate-to-severe at 2–4 months of age, we examined mice 3 to 3.5 months of age to determine if degeneration was occurring in tissues other than brain or spleen, including the kidney, liver and thymus. At this age, spleen size, cellularity and differential lymphocyte counts are comparable for wild-type and the *Atrn* mutants (data not presented). Kidney histology showed that the organization of individual nephrons seemed normal (Fig. 2a, c), but the interstitial matrix connecting the tubules was missing (Fig. 2b, d). Normal kidney stained well with PAS reagent (Fig. 2e, Additional file 1: Figure S1A) but not with Alcian blue (data not presented) indicating that the normal interstitial matrix contains substantial levels of glycosylated protein including proteoglycan but little acid mucopolysaccharide. In contrast, kidney from *Atrn* mutants did not stain well with PAS indicating reduced extracellular glycosylated material (Fig. 2f, Additional file 1: Figure S1b). Quantitative analysis assessed glycosylation as 31.1% across the control kidney section and 4.4% across the attractin-deficient kidney section (Supplemental Fig. 1c, d). Since the mutant signal coincided primarily with a strong haematoxylin nuclear signal, this is likely to be a background also for the control section. In contrast to the control, attractin-deficient kidney samples exhibited an almost complete loss of the extracellular matrix between the tubules, as well as similar loss of the proteoglycan-rich brush border within the tubules (Fig. 2e, f). There is not a generalized effect upon glycosylation within the mutant cells. Glycosylation of secreted proteins such as agouti destined for the extracellular compartment appears normal in *Atrn*^mg−3J/mg−3J^ mice [5]. Although a role for attractin in regulating specific glycosyltransferases cannot be excluded, the observed defects are consistent with a fault in extracellular matrix/proteoglycan secretion and deposition. In support, in the liver we find that the basement membrane between hepatocytes of wild-type mice is heavily stained (Fig. 3a, c) but the ECM between the hepatocytes of *Atrn*^mg−3J/mg−3J^ mice is strongly reduced (Fig. 3b, d). Note the absence of glycogen in the mutant hepatocytes, probably consequent to the higher basal metabolic rate associated with the neurodegeneration-induced tremor [7]. Using PAS staining, in contrast to the control condition (Fig. 3e, Additional file 1: Figure S2A), the liver of *Atrn*^mg−3J/mg−3J^ also appears to be relatively deficient in glycosylated protein (Fig. 3f, Additional file 1: Figure S2B). Quantitative analysis assessed glycosylation as 26.7% across the control liver section and 10.0% across the attractin-deficient liver section (Additional file 1: Figure S2C, D). For the mutant liver samples, the positive signal represented predominantly a background high nuclear staining by haematoxylin. Further, the sinuses are swollen with reduced presence of the ECM-dependent space of Disse (perisinusoidal space) (Fig. 3e, f). In the thymus, where we believe attractin plays little role based on mRNA expression [1], there is no observable histological difference between control and mutant animals (data not shown).

### Discussion

We have been unable to demonstrate any interaction of either natural or recombinant attractin ectodomain with any component of the ECM, and attractin does not appear to be a component of the ECM. We propose that attractin functions in proteoglycan-rich granule exocytosis and ECM maintenance in the differentiated state, a process that will replenish the plasma membrane with new membrane as exocytosis occurs. Given its location in electron-dense granule-rich secretory vesicles [1], attractin may have evolved a secondary function for aiding transport to the exterior of positively charged peptides including agouti and certain chemokines. The reduction or absence of ECM-proteoglycan would have profound effects upon the presentation of basic peptides and chemokines that may account in part for the immune and pigmentation-related functionality of attractin [13, 14]. The proposed role for vesicular trafficking attractin affecting ECM deposition and plasma membrane maintenance provides a unifying hypothesis for the pleiotropic effects of the null genotype and identifies avenues for further exploration. An additional consideration is that as yet unclassified human pathologies that involve neurodegeneration and concomitant renal dysfunction might be examined for abnormalities either at the *ATRN* locus or else its transcriptional control [15].

## Limitations

Since these results describe pathology associated with a mutation in the *Atrn* gene, the only limitation concerns genetic penetrance. We have observed these results in the two most severe *Atrn* mutations (*Atrn*^mg−3J/mg−3J^ and *Atrn*^mg−6J/mg−6J^) but have not examined the *Atrn*^mg/mg^ and *Atrn*^mg−L/mg−L^ variants, strains with less severe effects upon levels of normal *Atrn* transcript [7].

## Supplementary information

**Additional file 1: Figure S1.** Quantification of glycoprotein in wild-type and attractin-deficient kidney (PAS stain). **Figure S2.** Quantification of glycoprotein in wild-type and attractin-deficient liver (PAS stain).

## Data Availability

Data sharing is not applicable to this article as no datasets were generated or analysed during the current study.

## Abbreviations

α-MSH: α-Melanocyte Stimulating Hormone
CNS: Central Nervous System
ECM: Extracellular Matrix
H&E: Haematoxylin and Eosin
ACUC: Animal Care and Use Committee
Mc1R: Melanocortin-1 Receptor
PAS: Periodic Acid-Schiff reagent
RGB: Red/Green/Blue
