# Supplementary material for: Post-developmental extracellular proteoglycan maintenance in attractin-deficient mice
Source: BMC Res Notes. 2020 Jun 24;13:301. doi: 10.1186/s13104-020-05130-1 (PMC7313179; doi:10.1186/s13104-020-05130-1)
Supplement: Supplementary file 1 — Additional file 1: Figure S1. Quantification of glycoprotein in wild-type and attractin-deficient kidney (PAS stain). Figure S2. Quantification of glycoprotein in wild-type and attractin-deficient liver (PAS stain). [file 13104_2020_5130_MOESM1_ESM.docx]

**
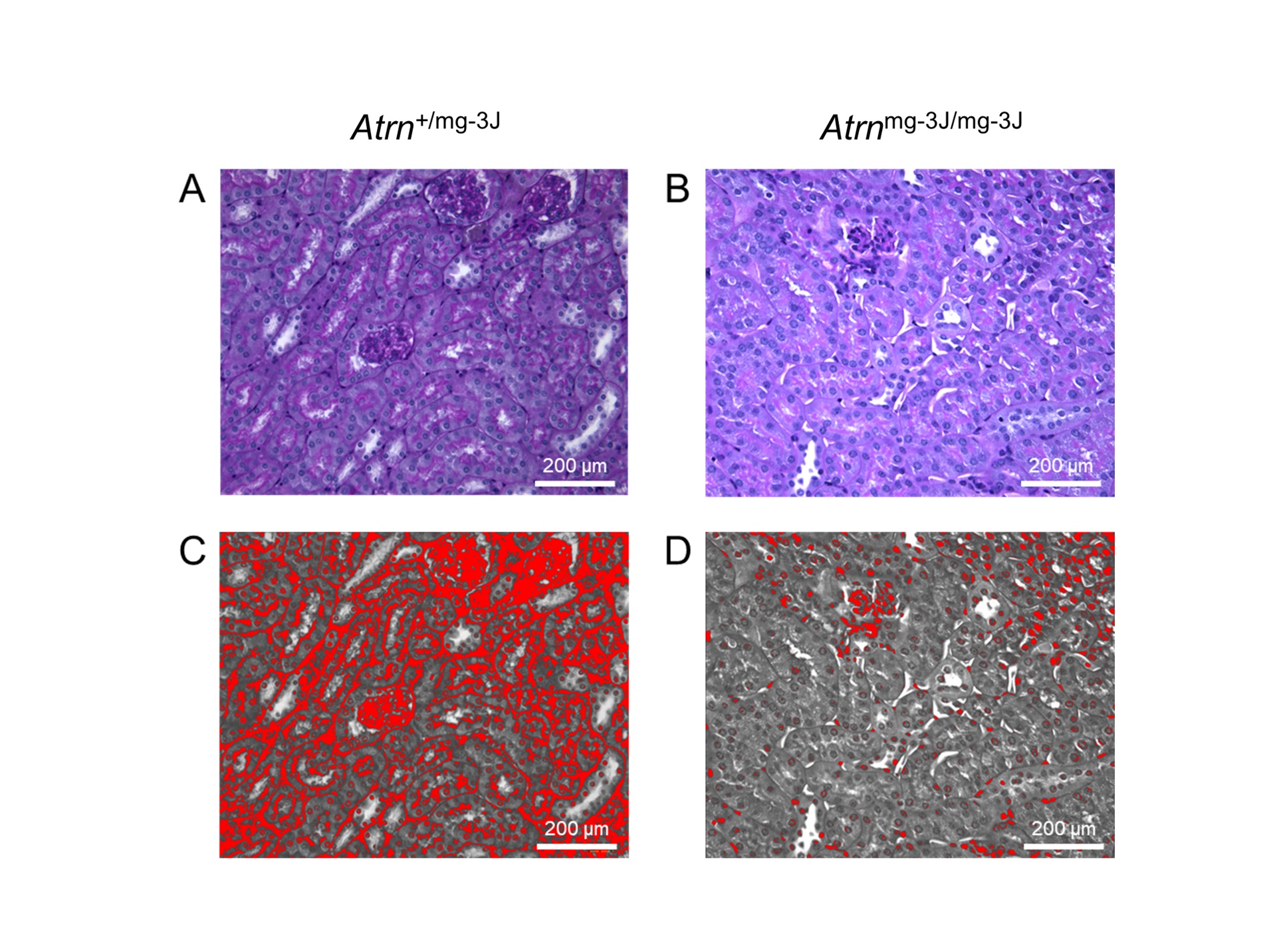
Additional Figure S1.**

A. Normal kidney and B. *Atrn*^mg-3J/mg-3J^ kidney (PAS stain). C. and D. Threshold analysis for PAS staining for the control kidney (A.), and the *Atrn*^mg-3J/mg-3J^ kidney (B.).

**Additional Figure
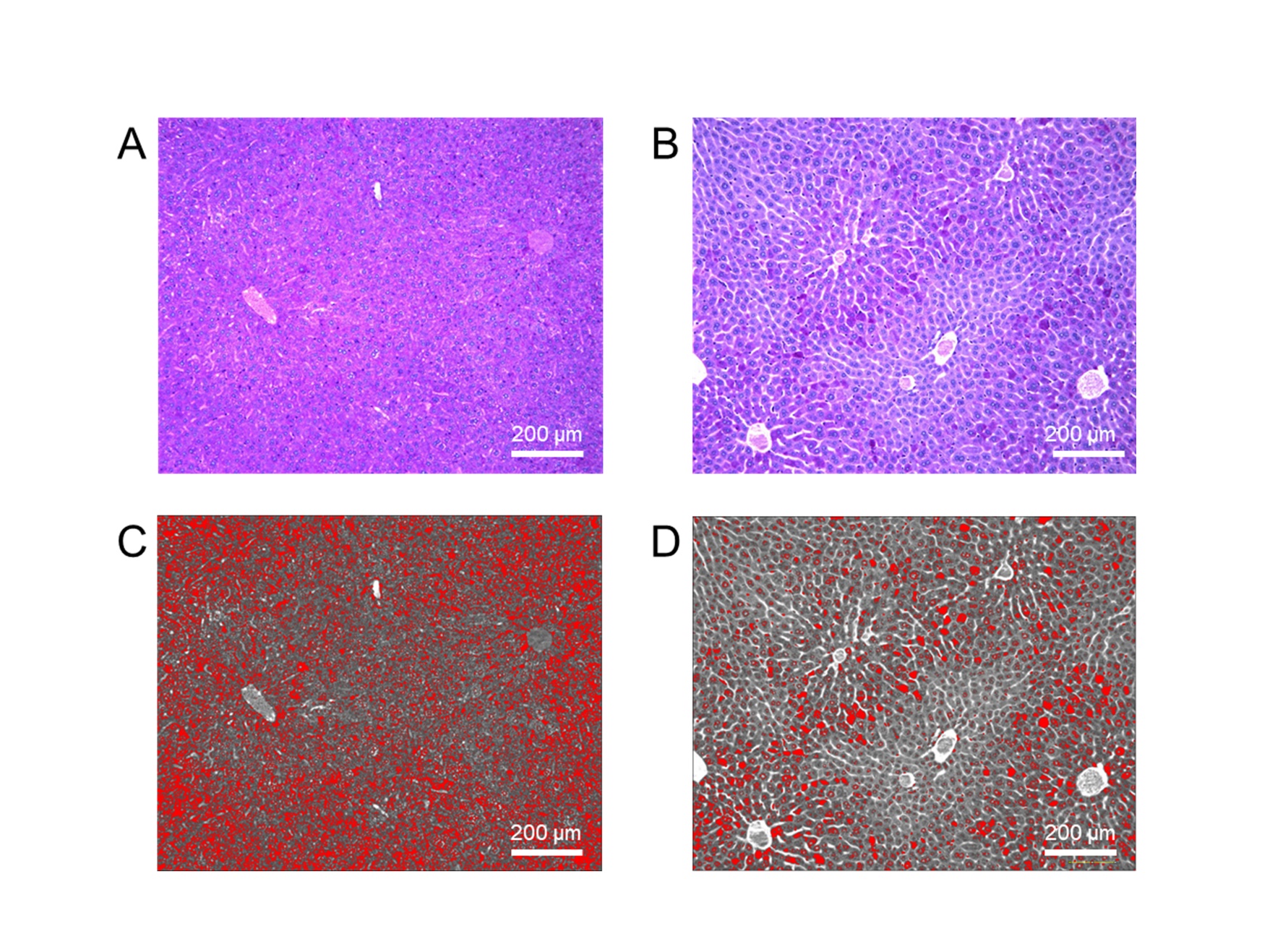
S2.**

A. Normal liver and B. *Atrn*^mg-3J/mg-3J^ liver (PAS stain). C. and D. Threshold analysis for PAS staining for the control liver (A.), and the *Atrn*^mg-3J/mg-3J^  liver (B.).
